# The data-driven decision-making, sustainable value creation, and international firm performance: Micro-level evidence based on AI language models

**DOI:** 10.1371/journal.pone.0340731

**Published:** 2026-02-11

**Authors:** Miao Xu, Bing Lu

**Affiliations:** School of Economics and Statistics, Zhejiang Gongshang University Hangzhou College of Commerce, Hangzhou, Zhejiang, China; East China Normal University, CHINA

## Abstract

Data-driven decision-making (DDDM) has become integral to managerial and organizational processes in the era of digitalization and internationalization. This study explores the impact of DDDM on international firm performance. Leveraging AI language models, specifically BERT and ChatGLM2-6B, to quantify DDDM, we find that DDDM positively impacts international firm performance. To uncover the mechanisms underlying this correlation, we develop a framework explaining how DDDM creates sustainable value for firms, thereby enhancing international firm performance across four dimensions: pollution prevention (current internal), green innovation (future internal), sustainability information disclosure (current external), and sustainability vision co-creation (future external). Additionally, this study reveals that the positive impact of DDDM on international firm performance is amplified by higher market competition, greater foreign shareholding, and state ownership.

## 1. Introduction

In recent years, the digital economy has emerged as a pivotal catalyst for economic and industrial transformations worldwide, reshaping how businesses operate and compete [1]. Digital technologies, such as the Internet of Things, big data, cloud computing, and artificial intelligence, are transforming firm performance in global markets by redefining cross-border business processes and reshaping profit distribution across regions. These transformative changes underscore the importance of understanding how the application of digital technologies (usually termed as digitalization) impacts international business.

As a key capability enabled by digitalization, data-driven decision-making (DDDM) has attracted increasing attention for its potential to enhance decision accuracy through systematic data analysis [2]. However, debates persist regarding how to effectively measure DDDM and evaluate its actual impact on firm performance. While some studies use case-based approaches to explore the theoretical implications of DDDM [3], others rely on survey-based indicators [2]. These methods often suffer from limited sample sizes and potential subjectivity, highlighting the need for more robust and scalable approaches to measuring DDDM.

This study addresses this challenge by introducing a novel methodology that leverages advanced AI language models, including BERT and ChatGLM2-6B, to construct a firm-level DDDM index based on text data from the Management Discussion and Analysis (MD&A) sections of firms’ annual reports. Using this index, we analyze the relationship between DDDM and international firm performance within the theoretical framework of sustainable value creation [4]. This framework emphasizes the creation of internal and external sustainable value, both in the short and long term, as a source of firm-specific advantages in international markets.

Empirically, this study examines a dataset of 2,873 Chinese listed firms from 2007 to 2022, offering insights into how DDDM adoption influences international firm performance. China, as the world’s second-largest economy, provides a compelling context for exploring the broader implications of DDDM, given its prioritization of the digital economy as a national strategic priority [5]. Our findings confirm that DDDM enhances firms’ sustainable value and international competitiveness, contributing to the ongoing discourse on the strategic role of digitalization in the global economy.

By integrating DDDM into the sustainable value framework, this study not only advances the understanding of DDDM’s role in international business but also provides a scalable tool for assessing its adoption and impacts. The remainder of this paper is organized as follows. Section 2 reviews the relevant literature. Section 3 theoretically analyzes the relationship between DDDM and international firm performance, as well as the underlying mechanisms. Section 4 introduces the data, variable measurement, methodology and model specifications. Section 5 presents and discusses the empirical results, while Section 6 concludes this study.

## 2. Literature review

### 2.1. Digitalization and international firm performance

Various theories have been widely applied to explain how digital technologies improve firm performance in international markets. According to the transaction cost theory, digital platforms and tools help reduce information asymmetry, lower production and coordination costs, and facilitate cross-border operations [6–10]. Resource-based theory emphasizes digital capabilities such as data analytics, knowledge sharing, and virtual integration as sources of firm-specific advantages and dynamic capabilities [11–13]. These perspectives offer useful insights into the mechanisms through which digitalization contributes to international competitiveness. Recent studies further extend these perspectives by highlighting the distinct characteristics of digital platform firms and data-driven practices in international business. Ojala et al. (2018) [14] argue that the internationalization process of digital ventures differs significantly from traditional firms due to novel business models and platform architectures. Zeng et al. (2019) [15] emphasize that internal resources alone are insufficient for platform firms in foreign markets, highlighting the need for leveraging external networks and real-time data. These developments underscore the importance of examining DDDM in international business.

### 2.2. Sustainable value and international firm performance

In the context of an increasingly pronounced global pursuit of sustainable development, sustainability practices have been confirmed as crucial determinants for firms engaging in international business [16,17]. Several important reasons help explain why sustainable value is associated with international firm performance. First, from the perspective of international competition, the increase in sustainable value enables firms to gain competitive advantages in international markets [18–20]. Firms that hesitate to adopt sustainable practices inevitably face pressure the competitive pressures exerted by sustainability-oriented competitors [21]. Second, from the perspective of investors and shareholders, firms with high sustainable value are considered relatively reliable [22], and are able to access external financial resources (e.g., debt) at relatively low cost [19]. Third, from the perspective of host countries, the increase in sustainable value helps firms establish good relationships with host governments, thereby gaining continuous financial and political supports [23]. For instance, environmental protection taxes implemented by host countries allow firms that adopt sustainable development strategies to reduce tax burden [24]. Additionally, the implementation of sustainable strategies enhances internal controls, reducing the potential litigation risks in overseas markets [25], thereby promoting a steady growth.

It is worth noting that implementing sustainable strategies is not easy, as it involves significant implementation costs [26]. Research has found that in practice, some “greenwashing” or “window-dressing” firms, despite poor environmental performance, create a superficial strategic image that misleads outsiders into believing they are genuinely practicing sustainable development strategies. This approach aims to gain the benefits of sustainable strategies without bearing the costs of implementing them [27,28].

Nevertheless, relevant discussion on how digitalization or DDDM affects international firm performance remains insufficient. Firstly, the methods used to measure digitalization or DDDM exhibit some limitations. Specifically, the keyword text-searching method fails to capture the semantics of sentences and paragraphs, often leading to misjudgments and omissions. For example, when examining annual reports of listed firms, we found that a significant portion of digital-related keywords appeared in sections on industry background or future development plans, which do not necessarily reflect digitalization practices. Consequently, we highlight the limitations of directly extracting keywords without considering their specific semantics and propose addressing this issue with more nuanced approaches. Secondly, there is limited empirical evidence regarding the influence of DDDM on international firm performance due to data availability constraints, as DDDM is difficult to measure accurately. Moreover, understanding the potential mechanisms by which DDDM affects international firm performance is crucial. Existing studies remain fragmented and lack a theoretical framework to systematically summarize these mechanisms. In this study, we adopt a quantitative method to measure the extent of firms’ DDDM practices, using AI language models. We further highlight the ways in which DDDM enables value creation and provide empirical evidence from Chinese listed firms.

## 3. Theoretical framework and hypotheses

### 3.1. The effects of DDDM on international firm performance

DDDM extends beyond the mere utilization of digital technologies; it involves the strategic use of data and advanced analytics to guide managerial decisions and organizational behaviors [29]. DDDM provides firms with rational decision-making solutions across stages, including R&D, production, management and marketing [30], which could benefit firms’ competencies, such as entrepreneurial orientation, international knowledge, learning capability, network, marketing capability, and innovation capability [31]. Recent developments in generative AI have further amplified the potential of DDDM by enhancing firms’ ability to extract insights, model future scenarios, and explore non-traditional strategies [32]. Furthermore, it allows organizations to transcend the limitations of conventional algorithms and human intuition by supporting the exploration of non-traditional, innovative solutions, while also reinforcing predictive analytics and strategic planning processes [33]. In operational domains, such as supply chain management and benchmarking operations, generative AI contributes to more informed decision-making, ultimately improving firm competitive advantages [34]. Additionally, generative AI empowers entrepreneurs to innovate business models by enabling decisions that were previously difficult to make [35]. Thus, DDDM offers a novel perspective to understanding how digitalization translates into international firm performance.

DDDM facilitates firms in accessing and expanding overseas markets. Using predictive algorithms to evaluate a firm’s current status and forecast its market potential, DDDM can optimize decision-making and accelerate internationalization [36]. By using digital platforms and ecosystems, DDDM serves as an approach to internationalization, accumulating knowledge and relationships, and creating value for global customers [37]. AI readiness, as a form of cognitive technology supporting DDDM, helps organizations optimize investment decisions and select diversified growth paths in resource-constrained contexts, thereby boosting market performance for small and medium-sized firms (SMEs) [38]. It is worth noting that DDDM is particularly important for SMEs, as it helps reduce barriers, such as time, resources, information and network constraints, thereby promoting their participation in the international market [38–40]. DDDM can help female-led SMEs improve the efficiency of market intelligence in the internationalization process, allowing entrepreneurs to better understand customer needs, market trends, and competitor dynamics, thereby making wiser decisions [41]. Advanced data-mining techniques, such as machine learning and statistical approaches, support firms in strategically modeling and interpreting data, and underpin the market-oriented strategies of most SMEs through accessible information sources [39]. Therefore, we propose the following hypothesis:

**Hypothesis 1.** DDDM has a positive effect on international firm performance.

### 3.2. The mechanism of sustainable value creation

**Fig 1:**
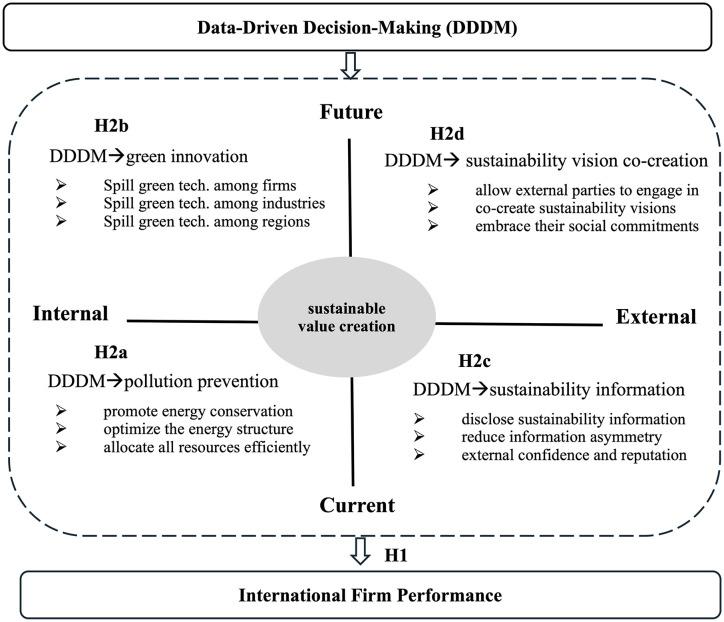
The mechanism of sustainable value creation.

We argue that sustainable value creation can be a crucial mechanism for explaining how DDDM promotes international firm performance. We analyze how DDDM creates sustainable value across four dimensions: current internal, future internal, current external, and future external. Fig 1 illustrates the sustainable value creation framework of DDDM, which is based on the model proposed by Hart and Milstein (2003) [4].

Firstly, from the current internal dimension, DDDM enables firms to minimize waste emissions during production and operations, effectively preventing pollution issues [1], thus generating short-term internal sustainable value [4]. Specifically, DDDM, as an emerging management practice, can identify and resolve inefficiencies in production and operation processes, optimize energy consumption patterns, and promote energy conservation and pollution reduction [42,43]. With the widespread adoption of DDDM in management processes, inefficient and high-energy-consuming production procedures are gradually eliminated, reducing overall energy consumption [44], thereby improving firms’ green total factor productivity [45]. Meanwhile, DDDM contributes to the advancement of renewable energy [46], promotes the development of clean energy sources [47], and optimizes the energy structure [48]. DDDM improves the efficiency of resource utilization [49], and reduces emissions during production, operation, and service processes [1]. Therefore, we propose the following hypothesis:

**Hypothesis 2a.** DDDM increases firms’ sustainable value from the current internal dimension through pollution prevention, thereby improving international firm performance.

Secondly, from the future internal dimension, DDDM promotes the development of green technologies [1], driving green technology innovation and enabling firms to gain sustainable value internally in the long run [4]. Green technology innovation primarily refers to innovative activities related to energy conservation, emission reduction, pollution control, waste recycling, and green management [50]. At the firm level, DDDM significantly promotes green innovation [51], as firms adopting DDDM possess greater organizational flexibility and tend to integrate more proactively into global innovation networks, thereby more effectively keeping pace with international trends in cutting-edge green innovation [52]. The driving force behind green innovation comes not only from technological advancements of individual firms but also from the spillovers of green innovation among firms. For example, Industry 4.0 technologies, which leverage advanced digital technologies and big data analytics to drive data-based decisions, enhance the dissemination of information and accumulation of knowledge among firms, encouraging them to embrace open innovation practices, consequently fostering advancements in green technology innovation [53]. At the industry level, green technology innovation has positive spillover effects. When individual firms develop new green technologies, their competitors often adopt similar green technologies to maintain their market share and competitive position. Such green technology spillover spreads along the industry chain, benefiting firms at various stages and improving the overall level of green innovation in the industry [54]. Therefore, we propose the following hypothesis:

**Hypothesis 2b.** DDDM increases firms’ sustainable value from the future internal dimension through green innovation, thereby improving international firm performance.

Third, from the current external dimension, DDDM, as a kind of objective and relatively open decision-making approach, can increase information disclosure, thereby reducing information asymmetry between firms and external parties, enabling firms to gain sustainable value externally in the current period [4]. Information disclosure systems evolve accordingly and intrinsically with the advancement of digitalization, driving down communication costs and enhancing the transparency of information exchange [55]. This serves to extend interaction and reduce information asymmetry between firms and external parties, such as customers, suppliers, social organizations, governments, and the media [4]. As a result, firms leveraging DDDM accumulate increased external confidence from the public, enhancing corporate reputation. In the interaction between firms and external parties, disclosing the firms’ own sustainable development-related practices can integrate the needs of stakeholders, which in turn further encourages firms to adopt more sustainable practices to maintain ongoing external confidence and reputation [4]. Therefore, we propose the following hypothesis:

**Hypothesis 2c.** DDDM increases firms’ sustainable value from the current external dimension through sustainability information disclosure, thereby improving international firm performance.

Finally, from the future external dimension, DDDM enables firms to embrace their social commitments [56] and to co-create sustainability visions with external parties, thereby enhancing firms’ external sustainable value [4]. DDDM strengthens firms’ collective inclinations, thereby fostering the drive to meet their social responsibilities [57], mainly because the application of digital technologies in the process of DDDM changes how firms participate in collective practices [58]. Meanwhile, the openness and inclusiveness of DDDM encourage external parties to participate in firms’ decision-making, promoting socially responsible practices [59]. Research has found that some firms are leveraging digital platforms to co-create social responsibility initiatives (e.g., donations) with consumers, serving as an emerging form of digital marketing [60]. Some firms are leveraging digital training technologies (e.g., VR or AR) to improve employees’ job skills, which can be regarded as a socially responsible practice [57]. Crucially, firms adopting DDDM can swiftly address the values of social stakeholders during the collaborative co-creation of collective practices [57]. Therefore, we propose the following hypothesis:

**Hypothesis 2d.** DDDM increases firms’ sustainable value from the future external dimension through sustainability vision co-creation, thereby improving international firm performance.

## 4. Empirical model design

### 4.1. Regression models

Our empirical analysis explores how international firm performance responds to DDDM using the following baseline specification:


$${lnsales}_{it}=\alpha_{\text{0}}+\alpha_{\text{1}}{DDDM}_{it}+{\Sigma_{k}\text{X}}_{it}+\Sigma Firm+\Sigma Year+\Sigma Industry+\Sigma Province+\varepsilon_{it}$$ (1)

where *i* indexes firms, and *t* indexes years. The dependent variable is *lnsales* and the independent variable is *DDDM*. *X* is a set of control variables at the firm level to account for other factors that may affect international firm performance. ΣFirm, ΣYear, ΣIndustry, and ΣProvince represent firm, year, three-digit industry and province fixed effects, respectively. The key coefficient of interest is $\alpha_{\text{1}}$, estimated on *DDDM*, which indicates the relationship between DDDM and international firm performance. The standard errors are clustered at the firm level to alleviate unobserved heterogeneity.

We investigate mechanisms using the specification shown in Eq (2), which is designed to examine the relationship between DDDM and mechanism variables (*Mechanism*).


$${Mechanism}_{it}=\beta_{\text{0}}+\beta_{\text{1}}{DDDM}_{it}+{\Sigma_{k}\text{X}}_{it}+\Sigma Firm+\Sigma Year+\Sigma Industry+\Sigma Province+\varepsilon_{it}$$ (2)

where *Mechanism* denotes mechanism variables. The key coefficient of interest is $\beta_{\text{1}}$, estimated for *DDDM*, which captures the relationship between *DDDM* and mechanism variables. All of the control variables and fixed effects in the specification adhere to those used in the baseline specification.

This method of mechanism analysis is derived from the idea of mediation effect analysis. Although the idea of mediation effects has been widely adopted, many scholars have raised concerns about its potential endogeneity issues. In response, various approaches have been proposed to improve the reliability of mediation analysis. A common practice [61,62] is to propose one or several mediator variables whose relationship with the dependent variable is theoretically intuitive and temporally and logically proximate. Under these conditions, formal causal inference methods are unnecessary to assess the causal link from mediator variables to the outcome.

Furthermore, to investigate the moderating effects of firm characteristics, we select several independent variables, and add interaction terms between these variables and DDDM into the baseline specification, as shown in Eq (3).


$${lnsales}_{it}=\alpha_{\text{0}}+\alpha_{\text{1}}{DDDM}_{it}+\alpha_{\text{2}}{Moder}_{it}+\alpha_{\text{3}}{{DDDM}_{it}\times Moder}_{it}+{\Sigma_{k}\text{X}}_{it}+\Sigma Firm+\Sigma Year+\Sigma Industry+\Sigma Province+\varepsilon_{it}$$ (3)

where *Moder* denotes firm characteristic variables. All other variables and fixed effects are the same as the baseline specification. The key coefficient of interest is $\alpha_{\text{3}}$, estimated for the interaction term, which indicates whether the examined firm characteristics moderate the relationship between DDDM and firm performance.

### 4.2. Variable definitions and explanations

#### 4.2.1. The independent variable.

The independent variable is the DDDM index (*DDDM*), which measures the degree to which firms adopt DDDM. Existing research on DDDM demonstrates multidisciplinary characteristics, yet quantitative measurements primarily rely on questionnaire surveys [2,63,64]. To address the limitations of the questionnaire method, such as subjectivity and limited sample size, this study adopts a text classification approach based on AI language models and unstructured text data to quantify DDDM.

The DDDM index was constructed using 50,000 annual reports of Chinese listed firms (2007−2022). First, Python was employed to extract the Management Discussion and Analysis (MD&A) sections via localization algorithms, filter irrelevant content (e.g., financial tables) using keyword matching, and split overlength paragraphs into 1.13M coherent units. Second, 50,000 paragraphs were randomly sampled for manual annotation based on predefined criteria for identifying DDDM practices, yielding 3,000 positive samples (labeled 1) and 47,000 negative samples (labeled 0). To mitigate insufficient learning of positive sample features by LLMs, 3,000 negative samples were resampled with all positive samples to form a balanced 6,000-sample training set, wherein 1,000 pieces were allocated as the development set. Additionally, 5,000 units were randomly sampled as the test set from the total sample. Third, A cascading strategy for text classification was implemented: Stage 1 used BERT to screen 1.13 million paragraphs (98% recall on the test set), generating 70,000 class-1 candidate texts; Stage 2 employed ChatGLM2-6B—retrained with tailored prompts—to classify these texts, achieving 96.4% overall accuracy. This strategy demonstrated superior performance over a single model in both efficiency and precision. Finally, following academic conventions, the DDDM index was calculated as the natural logarithm of (1 + the number of class-1 paragraphs in each firm’s annual report).

#### 4.2.2. The dependent variables.

As for the dependent variables, we use the natural logarithm of sales revenue in overseas markets (*lnsales*) to proxy for international firm performance in the baseline regressions. Although firm performance is multidimensional, sales growth has been widely accepted as one of the proxy variables for firm performance [65]. Similar to prior work [66], we also use the natural logarithm of the number of related firms in overseas markets (*lnaffiliates*) as another proxy for international firm performance in the robustness test.

#### 4.2.3. The control variables.

As for the control variables, following prior work [67], we select a group of firm-level variables, including a firm’s net return on assets (*ROA*), the natural logarithm of the capital-labor ratio measured by dividing the total fixed assets by the number of employees (*lncapital*), total liabilities scaled by total assets (*liability*), shareholders’ equity divided by the market value of equity (*Book_Market*), the shareholding ratio of the five largest shareholders (*top5*), and the percentage change in domestic sales revenue (*Home_sales*). All continuous variables are winsorized at 1%.

### 4.3. Data and sample

Data used to measure DDDM index are sourced from annual reports of Chinese listed firms and these reports are collected from CNINF.COM. Data on Chinese listed firms are collected from the China Stock Market and Accounting Research Database (CSMAR) and Chinese Research Data Services Platform (CNRDS). Our sample comprises 2,873 A-share listed firms in China from 2007 to 2022. Listed firms that satisfied one of the following conditions were excluded: (1) were in finance-related industries; (2) were listed as ST and *ST; (3) had missing financial data; or (4) were established in the observed year.

Table 1 provides a comprehensive summary of variables in this study, including their labels, definitions, explanations and sources.

**Table 1:** Variable definitions and explanations.

Labels	Variables	Definitions & Explanations	Source
*DDDM*	Independent variables	DDDM measured with AI language models (BERT and ChatGLM2-6B) by this study, explained in section 4.2.1.	Annual reports
*lnword*		The alternative proxy of DDDM, the natural logarithm of the number of searched keywords, measured with Term Frequency Analysis	CNRDS
*lnpara*		The alternative proxy of DDDM, the natural logarithm of the number of paragraphs that contains searched keywords, measured with Term Frequency Analysis	CNRDS
*lnsales*	Dependent variables	The natural logarithm of sales revenue in overseas markets	CNRDS
*lnaffiliates*		The natural logarithm of the number of related firms in overseas markets	CNRDS
*ROA*	Control variables	Net return on total assets	CSMAR
*lncapital*		The natural logarithm of capital-labor ratio measured by dividing the total fixed assets by the number of employees	CSMAR
*liability*		Total liabilities scaled by the total assets	CSMAR
*Book_Market*		Shareholders’ equity divided by market value of equity	CSMAR
*Top5*		Shareholding ratio of the sum of the five largest shareholders	CSMAR
*Home_sales*		The percentage change in domestic sales revenue	CSMAR
*Monitor*	variables of mechanism pollution prevention	=1 if the firm is monitored as a major pollution firm, =0 otherwise	CSMAR
*Accidents*		=1 if the firm experiences a major pollution incident, =0 otherwise	CSMAR
*Illegal*		=1 if the firm experiences illegal environmental events, =0 otherwise	CSMAR
*ISO9001*		=1 if the firm has obtained the ISO9001certification, =0 otherwise	CSMAR
*ISO14001*		=1 if the firm has obtained the ISO14001 certification, =0 otherwise	CSMAR
*Green_Invention*	variables of mechanism green innovation	The number of green invention patents independently obtained, the natural logarithm taken	CNRDS
*Green_Utility*		The number of green utility model patents independently obtained, the natural logarithm taken	CNRDS
*Green_sum*		Green_utility + green_invention, the natural logarithm taken	CNRDS
*E*	variables of mechanism sustainability information	Environmental performance score	CNRDS
*S*		Social performance score	CNRDS
*G*		Governance performance score	CNRDS
*ESG*		Overall ESG score	CNRDS
*analyst*	variables of mechanism sustainability vision co-creation	Analyst attention, measured as the number of analysts (teams) tracking and analyzing the enterprise	CSMAR
*reported*		Report attention, measured as the number of research reports tracking and analyzing the enterprise	CSMAR
*positive*		Sentiment of forum posts, measured as the proportion of positive posts in the stock forum of the listed firms	CSMAR
*HHI_A*	variables of moderating effects	Calculate the market share of an individual company within its industry by using its main business revenue. When the value closes to 1, the market is close to a monopoly market.	CSMAR
*HHI_B*		Calculate the market share of an individual company within its industry by using its operating revenue. When the value closes to 1, the market is close to a monopoly market.	CSMAR
*foreign_share*		The ratio of shares owned by foreigners	CSMAR
*SOE*		=1 if the firm is state-owned, = 0 otherwise	CSMAR

### 4.4. Statistics and correlations

Table 2 shows descriptive statistics of the full sample of 22,117 observations. The independent variable, *DDDM*, is ranging from 0 to 2.9440, with a median of 0.6930, while the dependent variable, *lnsales*, ranges from 3.3420 to 14.8900 with a median of 10.1900. This indicates DDDM and international firm performance exhibit substantial variation in our sample.

**Table 2:** Summary statistics.

*Variables*	N	S. D.	Min	P50	Max
*lnsales*	22,117	2.2310	3.3420	10.1900	14.8900
*DDDM*	22,117	0.7400	0.0000	0.6930	2.9440
*ROA*	22,117	0.0592	−0.2180	0.0404	0.1980
*liability*	22,117	0.1990	0.0547	0.4060	0.8830
*lncapital*	22,117	0.9410	9.9140	12.5800	15.0500
*Book_Market*	22,117	0.1560	0.0571	0.3270	0.7770
*Home_sales*	22,117	0.5390	−0.5960	0.0992	3.4470
*top5*	22,117	15.0600	20.9200	54.0800	89.4300

Table 3 presents the Pearson correlation coefficients among the variables. We also test the variance inflation factor (VIF) to ensure the low multicollinearity among variables. The mean VIF is 1.23.

**Table 3:** Pearson correlation coefficients.

	*lnsales*	*DDDM*	*ROA*	*liability*	*lncapital*	*Book_Market*	*Home_sales*	*top5*
*lnsales*	1							
*DDDM*	0.0410	1						
*ROA*	0.0205	0.0542	1					
*liability*	0.3222	−0.0213	−0.3963	1				
*lncapital*	0.1522	−0.1382	−0.1025	0.1474	1			
*Book_Market*	−0.0590	0.0165	0.1001	−0.5156	0.0259	1		
*Home_sales*	−0.1201	0.0079	−0.0111	0.0459	−0.0899	−0.0643	1	
*top5*	0.0390	0.0366	0.2279	−0.1159	−0.0509	0.1668	−0.0021	1

## 5. Empirical results

### 5.1. Baseline results

Following Eq (2), we employ multi-dimensional fixed effects to estimate the impact of DDDM on international firm performance. Table 4 reports the baseline results. Column (1) shows the results estimated under two-way fixed effects without control variables. The coefficient on *DDDM* is 0.0408, statistically significant at the 5% level. Column (2) shows the results in the presence of the aforementioned control variables with firm and year fixed effects, and standard errors clustered at the firm level. The coefficient on *DDDM* is 0.0399, statistically significant at the 5% level. Column (3) includes industry and province fixed effects to control for industry and geographical factors that may influence international firm performance. The coefficient on *DDDM* is 0.0365, statistically significant at the 5% level. These results indicate that DDDM significantly improves international firm performance, supporting Hypothesis 1.

**Table 4:** The impacts of DDDM on international firm performance.

Variables	(1)	(2)	(3)
	***lnsales***	***lnsales***	***lnsales***
*DDDM*	0.0408**	0.0399**	0.0365**
	(0.0202)	(0.0195)	(0.0182)
*ROA*		2.7624***	2.6310***
		(0.2525)	(0.2423)
*liability*		2.1112***	2.1130***
		(0.2014)	(0.1963)
*lncapital*		0.0816**	0.0715**
		(0.0327)	(0.0321)
*Book_Market*		0.6377***	0.6147***
		(0.1360)	(0.1308)
*Home_sales*		−0.0648***	−0.0522**
		(0.0203)	(0.0206)
*top5*		−0.0048*	−0.0044*
		(0.0025)	(0.0024)
Constant	9.9624***	8.0137***	8.1352***
	(0.0132)	(0.4528)	(0.4474)
Year FE	Yes	Yes	Yes
Firm FE	Yes	Yes	Yes
Province FE	No	No	Yes
Industry FE	No	No	Yes
Observations	22,692	22,117	22,117
Adj-R^2^	0.8247	0.8330	0.8363

Standard errors are clustered at the firm level in parentheses. *** is significant at 1%; ** is significant at 5%; * is significant at 10%.

### 5.2. Endogeneity and robustness

This section conducts several tests to ensure the robustness of the baseline results. Tables 5 and 6 present the results of these tests.

**Table 5:** Endogeneity.

Variables	*(1)*	*(2)*
	***DDDM***	***lnsales***
*IV_DDDM*	0.5263***	
	(0.1455)	
*DDDM*		1.4696**
		(0.6011)
*ROA*	0.0927	2.1471***
	(0.1022)	(0.2393)
*liability*	0.1170	1.7537***
	(0.0746)	(0.1670)
*lncapital*	−0.0396***	0.1135***
	(0.0135)	(0.0364)
*Book_Market*	0.1403**	0.4154***
	(0.0641)	(0.1461)
*Home_sales*	−0.0081	−0.0335
	(0.0105)	(0.0261)
*top5*	0.0010	−0.0034*
	(0.0010)	(0.0019)
Year FE	Yes	Yes
Firm FE	Yes	Yes
Province FE	Yes	Yes
Industry FE	Yes	Yes
Kleibergen-Paap rk LM statistic		13.088***
Cragg-Donald Wald F statistic		17.515
		[16.38]
Observations	17,574	17,574

Standard errors in parentheses. *** is significant at 1%; ** is significant at 5%; * is significant at 10%.

**Table 6:** Robustness.

Variables	(1)	(2)	(3)	(4)	(5)
	***Alternative proxies of variables***			***2009-2022***	***PPML***
	***lnsale***	***lnsale***	***lnaffiliates***	***lnsale***	***lnsale***
*lnword*	0.0726***				
	(0.0201)				
*lnpara*		0.0742***			
		(0.0217)			
*DDDM*			0.0218**	0.0339*	0.0034*
			(0.0100)	(0.0179)	(0.0018)
*ROA*	2.5202***	2.5209***	0.0132	2.5437***	0.2653***
	(0.2469)	(0.2469)	(0.1048)	(0.2447)	(0.0247)
*LEV*	2.1029***	2.1021***	0.9001***	2.0206***	0.2181***
	(0.1953)	(0.1953)	(0.0854)	(0.2006)	(0.0200)
*lncapital*	0.0708**	0.0712**	0.0326**	0.0700**	0.0079**
	(0.0329)	(0.0328)	(0.0162)	(0.0323)	(0.0032)
*Book_Market*	0.6476***	0.6480***	0.3789***	0.5547***	0.0610***
	(0.1336)	(0.1336)	(0.0647)	(0.1315)	(0.0132)
*Home_sales*	−0.0559***	−0.0556***	−0.0085	−0.0509**	−0.0053**
	(0.0211)	(0.0211)	(0.0093)	(0.0210)	(0.0021)
*top5*	−0.0050**	−0.0050**	−0.0016	−0.0045*	−0.0005**
	(0.0025)	(0.0025)	(0.0012)	(0.0025)	(0.0002)
Constant	2.5202***	2.5209***	0.0132	2.5437***	0.2653***
	(0.2469)	(0.2469)	(0.1048)	(0.2447)	(0.0247)
Year FE	Yes	Yes	Yes	Yes	Yes
Firm FE	Yes	Yes	Yes	Yes	Yes
Province FE	Yes	Yes	Yes	Yes	Yes
Industry FE	Yes	Yes	Yes	Yes	Yes
Observations	20,944	20,944	22,117	21,267	22,117
Adj-R^2^	0.8369	0.8369	0.7415	0.8427	

Standard errors are clustered at the firm level in parentheses. *** is significant at 1%; ** is significant at 5%; * is significant at 10%.

#### 5.2.1. IV estimations.

In this subsection, we apply the Instrumental Variable (IV) method to address concerns about possible endogeneity, particularly omitted variable bias and reverse causality. Following the approach of the Bartik instrument [68], we first calculate the province-industry-year average of DDDM (except itself) in the previous term (*Avg_DDDM*). This *Avg_DDDM* is expected to correlate with a firm’s DDDM, but it will not directly affect a firm’s decision-making. Therefore, the potentially endogenous component of DDDM can be partially removed, reducing concerns about endogeneity. Next, we compute the national average growth rate of DDDM (*Delta_DDDM*), which is plausibly exogenous to individual firm behavior. Then, we multiply *Avg_DDDM* and *Delta_DDDM* to obtain the instrumental variable, *IV_DDDM*. We use a two-stage least squares approach (2SLS) to estimate Eq (2) with this instrument.

The results in the first and second stages of IV (2SLS) are reported in the first and second columns of Table 5. More specifically, the coefficient on *IV_DDDM* is significantly positive at the 1% level in the first stage, and the coefficient on *DDDM* is significantly positive at the 5% level in the second stage. This IV passes both the weak identification and under-identification tests: the Cragg-Donald Wald *F*-statistic (17.515) exceeds the Stock-Yogo critical value at the 10% level (16.38), and the *p*-value of Kleibergen-Paap rk LM statistic is statistically significant at the 1% level. The IV (2SLS) results support the baseline findings that DDDM is positively related to international firm performance.

#### 5.2.2. Alternative proxies of variables.

We conduct robustness tests on the selection of the proxy for the dependent variable to alleviate concerns that our baseline analysis may yield biased findings due to reliance on a single measure. Following the extant literature, we adopt two alternative proxies for DDDM measured by text-searching and sourced from CNRDS. This database has compiled over 100 digitization-related keywords to construct a firm-level digitization index. One alternative proxy for DDDM is the natural logarithm of the number of searched keywords (*lnword*), and the other is the natural logarithm of the number of paragraphs that contain these keywords (*lnpara*). Columns (1) and (2) of Table 6 report the results of regressions using these alternative measures of DDDM. We find that the coefficients on *DDDM* are significant at the 1% level. These results confirm that our baseline finding is robust to alternative proxies for DDDM.

Similarly, we consider the proxy for the independent variable. Following extant literature, we use the natural logarithm of the number of related overseas firms (*lnaffiliates*) as an alternative proxy for international firm performance. Column (3) of Table 6 reports the results of regressions where international firm performance is measured by the number of related overseas firms. We find that the coefficient on *DDDM* is still significant at the 5% level. Accordingly, the baseline findings hold with an alternative proxy for the dependent variable.

#### 5.2.3. Sample selection.

Next, we conduct robustness tests on sample selection to reduce concerns that the baseline results might depend on a specific sample period. In Column (4) of Table 6, we set 2009 as the starting year, given that 2008 was the year of the global financial crisis. The results are generally consistent with baseline findings, alleviating concerns that the baseline results might be driven by a particular sample period.

#### 5.2.4. Alternative method of regression.

Furthermore, we conduct robustness tests using an alternative estimation method to reduce concerns that the baseline results might depend on the specific regression method. We adopt the Poisson pseudo maximum likelihood (PPML) to re-estimate Eq (2). PPML is a variant of Poisson regression, suitable for non-negative dependent variables that include zero values [69], which is precisely the case in this study. Column (5) of Table 6 reports that the coefficient on *DDDM* is significant. Accordingly, the baseline findings hold under the alternative regression model.

### 5.3. Mechanism analysis

In this section, we test Hypotheses 2a to 2d and examine why DDDM is positively related to international firm performance.

#### 5.3.1. The mechanism of pollution prevention.

First, from the current internal dimension, we investigate whether DDDM can reduce pollution emissions and increase firms’ short-term sustainable value internally. Regression results are shown in Table 7. Column (1) reports the regression results with the mechanism variable, *Monitor*, which equals1 if the firm is monitored as a polluter and 0 otherwise. The results in Column (1) show that the coefficient on *DDDM* is negative and significant at the 1% level, which indicates that DDDM reduces the probability of being monitored as a polluter. Column (2) reports the regression results using *Accidents* as the mechanism variable, which equals 1 if the firm experiences a major environmental pollution incident and 0 otherwise. The results in Column (2) show that the coefficient on *DDDM* is negative and significant at the 5% level, which indicates that DDDM reduces the probability of environmental pollution incidents. Column (3) reports the regression results using *Illegal* as the mechanism variable, which equals 1 if the firm experiences illegal events related to environment protection and 0 otherwise. The results in Column (5) show that the coefficient on *DDDM* is negative and statistically significant at the 5% level, indicating that DDDM reduces the likelihood of environmental illegal events. Columns (4) and (5) report the regression results using *ISO9001* and *ISO14001* as the mechanism variables, which equal 1 if the firm has obtained the ISO9001 or the ISO14001 respective certification, and 0 otherwise. These results show that the coefficients on *DDDM* are both positive and significant at the 1% level, which indicates that DDDM facilitates firms’ attainment the environmental certifications.

**Table 7:** The mechanism of pollution prevention.

Variables	(1)	(2)	(3)	(4)	(5)
	***Monitor***	***Accidents***	***Illegal***	***ISO9001***	***ISO14001***
*DDDM*	−0.0202***	−0.0010**	−0.0039**	0.0193***	0.0291***
	(0.0053)	(0.0004)	(0.0015)	(0.0062)	(0.0060)
*ROA*	0.2073***	0.0096**	0.0100	−0.0044	0.0880
	(0.0603)	(0.0043)	(0.0176)	(0.0667)	(0.0680)
*liability*	0.0113	−0.0001	−0.0110	−0.0577	−0.0141
	(0.0413)	(0.0023)	(0.0085)	(0.0453)	(0.0436)
*lncapital*	0.0189**	−0.0004	−0.0006	−0.0135*	−0.0090
	(0.0074)	(0.0004)	(0.0013)	(0.0079)	(0.0076)
*Book_Market*	0.0716**	−0.0038*	−0.0130*	−0.0397	−0.0211
	(0.0345)	(0.0021)	(0.0079)	(0.0393)	(0.0369)
*Home_sales*	0.0008	0.0000	−0.0012	0.0048	0.0005
	(0.0051)	(0.0004)	(0.0011)	(0.0060)	(0.0055)
*top5*	0.0019***	−0.0000	0.0000	−0.0013**	−0.0011*
	(0.0005)	(0.0000)	(0.0001)	(0.0006)	(0.0006)
Constant	−0.1249	0.0090*	0.0282	0.5695***	0.4637***
	(0.0994)	(0.0050)	(0.0190)	(0.1094)	(0.1055)
Year FE	Yes	Yes	Yes	Yes	Yes
Firm FE	Yes	Yes	Yes	Yes	Yes
Province FE	Yes	Yes	Yes	Yes	Yes
Industry FE	Yes	Yes	Yes	Yes	Yes
Observations	21,774	21,774	21,774	21,774	21,774
Adj-R^2^	0.5210	0.0019	0.0698	0.4379	0.5210

Standard errors are clustered at the firm level in parentheses. *** is significant at 1%; ** is significant at 5%; * is significant at 10%.

The results in Table 7 indicate that the positive impact of DDDM on international firm performance is associated with its effect on pollution prevention. When the adoption of DDDM increases, firms tend to reduce waste and emissions, lower the incidence of environmental pollution events, and are more likely to obtain environmental certifications. Therefore, Hypothesis 2a is supported.

#### 5.3.2. The mechanism of green innovation.

Secondly, from the future internal dimension, we investigate whether DDDM can promote green innovation and increase firms’ long-term sustainable value internally. The mechanism of green innovation can be captured by the number of green invention patents independently obtained (*Green_Invention*), the number of green utility model patents independently obtained (*Green_Utility*), and the sum of these two patents (*Green_sum*). The results of regressions are presented in Table 8. The coefficients on *DDDM* are significantly positive in all three columns. DDDM not only promotes growth in firms’ green invention patents but also boosts the development of green utility model patents. The improvement in green innovation performance increases firms’ long-term sustainable value, strengthens their international competitiveness, and consequently enhances international firm performance. Therefore, Hypothesis 2b is supported.

**Table 8:** The mechanism of green innovation.

Variables	(1)	(2)	(3)
	***Green_Invention***	***Green_Utility***	***Green_sum***
*DDDM*	0.0441***	0.0240**	0.0484***
	(0.0117)	(0.0107)	(0.0128)
*ROA*	0.2242*	0.3949***	0.4199***
	(0.1166)	(0.1179)	(0.1425)
*liability*	0.3826***	0.4387***	0.5801***
	(0.0756)	(0.0766)	(0.0927)
*lncapital*	0.0217	0.0410***	0.0405**
	(0.0149)	(0.0147)	(0.0176)
*Bk_Mkt*	0.2348***	0.2275***	0.2997***
	(0.0639)	(0.0645)	(0.0781)
*Sales_grow*	0.0127	0.0079	0.0201*
	(0.0096)	(0.0098)	(0.0117)
*top5*	0.0026**	0.0008	0.0017
	(0.0011)	(0.0011)	(0.0013)
Constant	−0.1524	−0.2606	−0.1568
	(0.2061)	(0.2010)	(0.2391)
Year FE	Yes	Yes	Yes
Firm FE	Yes	Yes	Yes
Province FE	Yes	Yes	Yes
Industry FE	Yes	Yes	Yes
Observations	22,111	22,111	22,111
Adj-R^2^	0.6483	0.6353	0.6764

Standard errors are clustered at the firm level in parentheses. *** is significant at 1%; ** is significant at 5%; * is significant at 10%.

#### 5.3.3. The mechanism of sustainability information disclosure.

Thirdly, from the current external dimension, we investigate whether DDDM can encourage sustainability information disclosure and increase externally generated short-term sustainable value. The mechanism of sustainability information disclosure can be captured by ESG scores, which are typically assessed by external agencies that collect and analyze relevant data, and evaluate firms based on environmental, social and governance (ESG) criteria. Specifically, we select the following four variables as proxies: (1) environmental performance score (*E*); (2) social performance score (*S*); (3) governance performance score (*G*); and (4) overall ESG score (*ESG*). These variables are individually substituted as the mechanism variable in Eq (2). The regression results, reported in columns (1) to (4) of Table 9, show that the coefficients on *DDDM* are all significantly positive. This confirms that DDDM promotes the disclosure of sustainability information, thereby increasing the current sustainable value obtained externally by firms, aiding them in participating in international competition, and improving their international firm performance. Therefore, Hypothesis 2c is supported.

**Table 9:** The mechanism of sustainable information disclosure.

Variables	(1)	(2)	(3)	(4)
	***E***	***S***	***G***	***ESG***
*DDDM*	0.3505*	0.6024***	0.1913*	0.5255***
	(0.1956)	(0.1531)	(0.1158)	(0.1179)
*ROA*	3.2122	−1.6780	−1.7144	1.3928
	(2.3037)	(1.7628)	(1.3633)	(1.3951)
*liability*	−2.1035	0.2463	5.3235***	2.0346**
	(1.4788)	(1.1477)	(0.8042)	(0.8665)
*lncapital*	0.7261***	0.4213**	0.5991***	0.6302***
	(0.2669)	(0.2027)	(0.1608)	(0.1550)
*Book_Market*	−0.7573	0.6851	−4.3075***	−1.4513**
	(1.3389)	(0.9394)	(0.6980)	(0.7374)
*Home_sales*	−0.0739	0.3505**	−0.4926***	−0.0735
	(0.1903)	(0.1538)	(0.1346)	(0.1143)
*top5*	0.0394**	−0.0104	0.0355***	0.0148
	(0.0194)	(0.0150)	(0.0098)	(0.0114)
Constant	6.6176*	18.2352***	12.5234***	17.8809***
	(3.6486)	(2.7554)	(2.1331)	(2.0975)
Year FE	Yes	Yes	Yes	Yes
Firm FE	Yes	Yes	Yes	Yes
Province FE	Yes	Yes	Yes	Yes
Industry FE	Yes	Yes	Yes	Yes
Observations	21,878	21,878	21,878	21,878
Adj-R^2^	0.6319	0.4747	0.5503	0.6517

Standard errors are clustered at the firm level in parentheses. *** is significant at 1%; ** is significant at 5%; * is significant at 10%.

#### 5.3.4. The mechanism of sustainability vision co-creation.

Finally, from the future external dimension, we explore whether DDDM encourages sustainability vision co-creation and increases externally generated long-term sustainable value. To measure firms’ interaction with external stakeholders [70], we use the following three variables as proxies: (1) analyst attention *(analyst*), which counts the number of analysts (teams) tracking and analyzing the firm, reflecting the degree of interaction between the firm and investment analysts; (2) research report attention (*reported*), which counts the number of research reports tracking and analyzing the firm, reflecting the degree of interaction between the firm and research institutions; (3) sentiment of forum posts (*positive*), which measures the proportion of positive posts in the firm’s stock forum, reflecting the degree of interaction between the firm and retail investors. These variables are individually substituted as the *Mechanism* in Eq (2). The regression results, reported in columns (1) to (3) of Table 10, show that the coefficients on *DDDM* are significantly positive. This indicates that DDDM enhances firms’ interactions with analysts, research institutions, and the public, forming a virtuous cycle that increases future external sustainable value and promotes firms’ performance. Accordingly, Hypothesis 2d is verified.

**Table 10:** The mechanism of sustainability vision co-creation.

Variables	(1)	(2)	(3)
	***analyst***	***reported***	***positive***
*DDDM*	0.4117***	0.8105**	0.0014**
	(0.1465)	(0.3723)	(0.0006)
*ROA*	41.2331***	101.5730***	0.0797***
	(2.0923)	(5.4713)	(0.0065)
*liability*	−8.2946***	−17.3886***	−0.0066
	(1.1356)	(2.8859)	(0.0040)
*lncapital*	0.3283	0.9672**	−0.0006
	(0.2048)	(0.4694)	(0.0007)
*Book_Market*	−19.5640***	−51.5000***	−0.0298***
	(1.0299)	(2.6359)	(0.0040)
*Home_sales*	−0.2710**	−0.6115*	0.0010*
	(0.1361)	(0.3167)	(0.0006)
*top5*	0.0333**	0.0632*	0.0004***
	(0.0146)	(0.0363)	(0.0000)
Constant	12.1388***	24.3806***	0.2886***
	(2.8128)	(6.4914)	(0.0094)
Year FE	Yes	Yes	Yes
Firm FE	Yes	Yes	Yes
Province FE	Yes	Yes	Yes
Industry FE	Yes	Yes	Yes
Observations	15,590	15,726	17,122
Adj-R^2^	0.6172	0.6122	0.6324

Standard errors are clustered at the firm level in parentheses. *** is significant at 1%; ** is significant at 5%; * is significant at 10%.

### 5.4. Moderating effects of firm heterogeneity

Recent studies suggest that DDDM can be further contextualized within institutional theory, which highlights how decision-making practices are shaped by broader regulatory and normative environments [71,72]. In light of this, we examine the Herfindahl Index (*HHI*), the ratio of shares owned by foreigners (*foreign_share*), and the nature of firm ownership (*SOE*) as three proxies for institutional environments, which serve as the heterogeneity variables in this study. Table 11 presents the results of these regressions.

**Table 11:** Heterogeneity.

Variables	(1)	(2)	(3)	(4)
	***lnsales***	***lnsales***	***lnsales***	***lnsales***
*DDDM*	0.0638***	0.0651***	0.0233	0.0881***
	(0.0245)	(0.0245)	(0.0195)	(0.0213)
*DDDM* × *HHI_A*	−0.2275*			
	(0.1316)			
*DDDM* × *HHI_B*		−0.3149*		
		(0.1688)		
*DDDM* × *foreign*			0.0023*	
			(0.0012)	
*DDDM* × *SOE*				−0.1643***
				(0.0383)
*ROA*	2.6262***	2.6296***	2.5975***	2.7053***
	(0.2423)	(0.2424)	(0.2414)	(0.2452)
*liability*	2.1161***	2.1172***	2.1215***	2.1248***
	(0.1964)	(0.1964)	(0.1960)	(0.1980)
*lncapital*	0.0719**	0.0718**	0.0661**	0.0735**
	(0.0321)	(0.0321)	(0.0318)	(0.0325)
*Book_Market*	0.6160***	0.6170***	0.6132***	0.6278***
	(0.1310)	(0.1310)	(0.1302)	(0.1315)
*Home_sales*	−0.0527**	−0.0517**	−0.0516**	−0.0566***
	(0.0206)	(0.0206)	(0.0205)	(0.0205)
*top5*	−0.0044*	−0.0044*	−0.0073***	−0.0038
	(0.0025)	(0.0025)	(0.0026)	(0.0025)
Constant	8.1179***	8.1121***	8.2739***	7.9912***
	(0.4500)	(0.4487)	(0.4464)	(0.4481)
Year FE	Yes	Yes	Yes	Yes
Firm FE	Yes	Yes	Yes	Yes
Province FE	Yes	Yes	Yes	Yes
Industry FE	Yes	Yes	Yes	Yes
Observations	22,097	22,111	22,117	21,695
Adj-R^2^	0.8364	0.8364	0.8373	0.8371

Standard errors are clustered at the firm level in parentheses. *** is significant at 1%; ** is significant at 5%; * is significant at 10%.

First, the relationship between DDDM and international firm performance can be affected by the level of market competition (*HHI*). DDDM enables firms to maintain competitive advantages, which helps firms expand overseas markets at a relatively low cost. As expected, when the competition in a market becomes intensifies, the influence of DDDM is expected to be stronger. To examine whether the positive impact of DDDM on international firm performance is strengthened as market competition increases, we add interaction terms between *DDDM* and *HHI* into the baseline specification. We choose two Herfindahl indexes (*HHI_A* and *HHI_B*) as proxies for the level of competition in a market, and report the results in the first and second columns of Table 11. As expected, the results show that the estimated coefficients of the interaction terms are negative and significant. In other words, as the Herfindahl index decreases and the market competition becomes more intense, the positive relationship between DDDM and international firm performance is reinforced.

Second, the role of the ratio of shares owned by foreigners (*foreign_share*) should be particularly considered in a study relevant to international firm performance. The interaction term between *DDDM* and *foreign_share* is introduced into the baseline specification and the result is shown in Column (3) of Table 11. The results show that the estimated coefficient of the interaction term is significantly positive, indicating that as the proportion of foreign shareholding increases, the impact of DDDM on international firm performance also increases. This suggests that firms with a higher proportion of foreign shareholding are better able to leverage sustainable value, thereby improving their international firm performance.

Third, the nature of firm ownership (*SOE*) is especially relevant to the research of the aforementioned mechanism, which could be captured by the dummy variable of *SOE* that equals 1 if the firm is state-owned and 0 otherwise. The interaction term between *DDDM* and *SOE* is introduced into the baseline specification and the result is shown in Column (4) of Table 11. The coefficient on the interacted term is negative and significant, which evidences that the fact that firms are state-owned can weaken the impact of DDDM on international firm performance is weaker when firms are state-owned, compared to non-state-owned firms. One possible explanation is that state-owned firms are relatively less affected by external environments or social shareholders, thereby reducing the necessity of increasing sustainable value and diminishing the impact of DDDM on international firm performance. Therefore, DDDM is more conducive to overcoming the challenges of international firm performance faced by non-state-owned firms.

## 6. Conclusion and discussion

### 6.1. Conclusion

This study introduces AI language models to quantify DDDM, laying an important foundation for subsequent research on digital management and digital economy. By leveraging two AI language models, BERT and ChatGLM2-6B, to conduct the text classification tasks, we effectively identify DDDM practices in annual reports of listed firms, and develop a DDDM index.

Furthermore, this study attempts to complement the current literature by highlighting the importance of DDDM to international firm performance. We explore the relationship between DDDM and international firm performance, using firm-level panel data. The sample includes about 2,873 listed firms in China and spans the period of 2007–2022. We start our empirical analysis by investigating international firm performance as a function of DDDM and other firm-level variables with the time, firm, province, and industry fixed effects. We find that DDDM is positively associated with international firm performance. Next, we attempt to answer why DDDM affects international firm performance by exploiting the mechanisms. Our theoretical framework, inspired by Hart and Milstein (2003) [4], underscores how DDDM creates sustainable value and promotes international firm performance in four dimensions. Specifically, DDDM has pollution prevention effects, green innovation effects, sustainability information disclosure effects, and sustainability vision co-creation effects in the dimensions of current internal, future internal, current external, and future external, respectively. This enhances firms’ competitive advantages in international markets, thereby improving international firm performance. Finally, we examine how firms’ characteristics moderate the effects of DDDM on international firm performance. The positive impacts of DDDM on international firm performance is enhanced when the level of market competition is higher, the proportion of foreign shareholding is higher, and the firm is state-owned.

### 6.2. Limitations and future research

This study primarily focuses on Chinese listed firms, which may not fully capture the economic impacts of DDDM on firm performance. The exclusion of small and medium-sized enterprises from the research sample is a limitation. Future research should focus on the full range of firms to provide a more general understanding of the effects of DDDM on firm performance across firm sizes. In addition, recent development of AI technology is likely to increases the application scope of DDDM; however, despite all these promising developments, caution remains that human intelligence should not be overlooked. Future research should focus on the comparison of AI-based and human-based decision-making to find out how to optimize the decision-making in practice. Future studies could also attempt preliminary experiments that integrate the proposed framework with emerging AI advances to further enrich the understanding of DDDM in practice.
